# Influence of Environmental Factors on the Growth of *Colletotrichum godetiae*, Causal Agents of Olive Anthracnose in Spain

**DOI:** 10.1111/1758-2229.70276

**Published:** 2026-02-12

**Authors:** Anabel Expósito‐Díaz, Angel Medina, Juan Moral

**Affiliations:** ^1^ Department of Agronomy, Campus of Rabanales University of Cordoba Cordoba Spain; ^2^ Magan Centre of Applied Mycology Cranfield University Cranfield UK; ^3^ Competitive Research Unit on Zoonoses and Emerging Diseases (ENZOEM) University of Cordoba Cordoba Spain

**Keywords:** climate change, *Colletotrichum* spp., fungal growth conditions, olive anthracnose

## Abstract

Olive anthracnose, the most critical disease affecting fruit and oil quality, is caused by *Colletotrichum* species, with *C. godetiae* dominant in Italian and Spanish orchards. Climate change could exacerbate its impact, particularly through rising temperatures and altered CO_2_ levels. This study evaluated the growth of two *C. godetiae* strains (Col‐558 and Col‐493) on Potato Dextrose Agar (PDA) and ‘Picudo’ fruit‐agar medium in different assays. Firstly, the strains were incubated at 10°C, 20°C, 25°C and 35°C and CO_2_ concentrations of 400 and 1000 ppm. Secondly, both strains were grown at the same temperatures but at different water activity (aw) levels (0.90, 0.94 and 0.98). Temperature was the main factor affecting fungal growth, explaining 64.2% of the variance in lag time and growth rate; CO_2_ had no significant effect. Conversely, aw and temperature significantly influenced mycelial growth, contributing 38.0% and 34.2% of the variance, respectively. An aw of 0.90 consistently resulted in a lag time exceeding 14 days across all temperatures. Furthermore, no growth was observed at 35°C under any aw or media conditions. These findings enhance our understanding of the environmental constraints on *C. godetiae* growth, highlighting the need for further research to develop mitigation strategies for olive anthracnose.

## Graphical abstract

Growth of *Colletotrichum godetiae* was mainly driven by temperature and water activity, while elevated CO_2_ showed no significant effect.

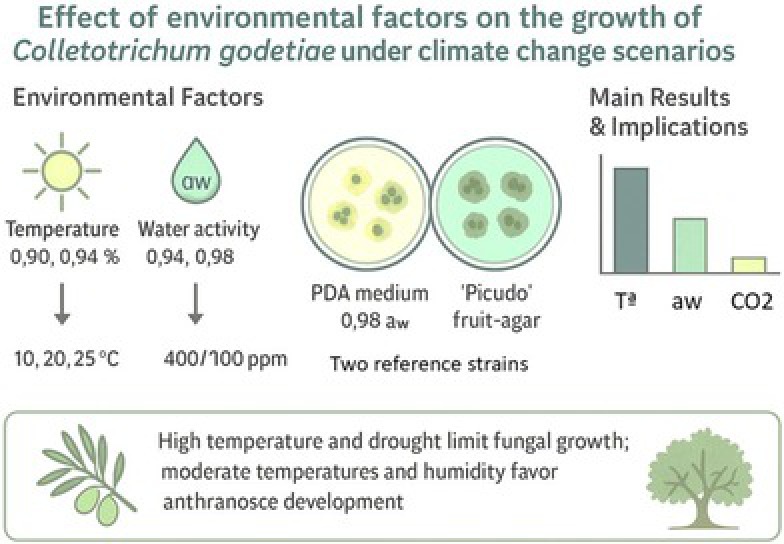

## 1 Introduction

The olive tree ( *Olea europaea* L) crop has experienced significant growth in recent decades, covering over 10 million ha worldwide (FAO 2024). As the most extensive perennial crop globally, olive cultivation is deeply rooted in the Mediterranean Basin, where the leading producers are Spain, Italy, Greece, and Portugal. Olive fruits are used to extract extra virgin olive oil but are also consumed as table olives; both are essential components of the traditional Mediterranean diet (Foscolou et al. 2018; Lucena et al. 2017).

Aerial fungal pathogens cause economically significant diseases in all olive‐growing regions (Agosteo et al. 2023). Among these pathogens, *Colletotrichum* species cause Anthracnose, the most critical olive fruit disease (Moral et al. 2009; Talhinhas et al. 2018). Olive anthracnose is endemic to the western Mediterranean Basin but has spread to all continents (Talhinhas et al. 2018). Under favourable environmental conditions, *Colletotrichum* species can cause severe fruit rot, resulting in devastating losses in fruit production. Even low levels of fruit‐rot incidence can compromise the organoleptic and nutritional quality of olive oil, reducing its market value (Leoni et al. 2018).

The Mediterranean region is particularly vulnerable to climate change and is identified as a hot spot for the 21st century (Lionello and Scarascia 2018; Orlowsky and Seneviratne 2012).

Regional feedbacks amplify warming, leading to 2.2°C–3°C increases in daily maximum temperature (Seneviratne et al. 2016; Vogel et al. 2021). Additionally, the global concentration of CO_2_ has been rising by approximately 20 ppm per decade since 2000, a rate that is 10 times faster than any sustained rise in CO_2_ during the past 800,000 years (IPCC 2019; Lüthi et al. 2008). Global warming also affects the soil water content, leading to significant reductions in winter and spring (Samaniego et al. 2018).

Experimental studies suggest that the projected rise in atmospheric CO_2_ levels over the next 25–50 years could exacerbate the impact of plant diseases (Magan et al. 2011; Medina et al. 2017; Raza and Bebber 2022). In olive crops, previous studies on diseases such as scab (*Fusicladium oleagineum*) or Verticillium wilt (*Verticillium dahliae*) have demonstrated that increased temperatures and changes in water availability can aggravate the severity of diseases (Calderón et al. 2014; Habbadi et al. 2023). However, specific information about olive anthracnose remains limited. Water stress, precipitation, water activity (aw), and evapotranspiration are interconnected factors that influence fungal diseases in olive crops. Adequate precipitation can prevent water stress but also facilitates the dispersion of *Colletotrichum* spores and prolongs the wetness of fruit surfaces, promoting the disease (Moral and Trapero 2012; Moral et al. 2014). Conversely, olive fruits cultivated under water stress show greater resistance to *Colletotrichum* infection (Conde‐Innamorato et al. 2025). However, while water availability from irrigation influences water content on fruit, it does not directly correlate with surface aw, which is also shaped by factors like waxes and cuticular structures on the fruit surface (Barbosa‐Cánovas et al. 2007).

Researchers have used water activity (aw) to simulate pathogen growth under various climate scenarios (Cervini et al. 2020; Medina et al. 2017). Fungal species are classified based on their response to aw: hydrophilic fungi thrive at levels above 0.90 aw, mesophilic fungi prefer levels between 0.85 and 0.90 aw, and xerophilic fungi exhibit optimal growth between 0.65 and 0.85 aw (Almiman 2024). Understanding these thresholds is essential for predicting how climate change and water availability will influence the epidemiology of *Colletotrichum* and other fungal pathogens in olive crops.

Weather conditions also impact the physiology and productivity of olive crops. For example, a 4°C increase in temperature can reduce olive fruit yield while altering its characteristics and maturation. Additionally, olive fruit maturation is advanced and extended in trees in warmer temperatures (Benlloch‐González et al. 2019). An early and prolonged ripening period is a well‐known risk factor for olive anthracnose, as ripe fruit is significantly more susceptible to the pathogen (Miho et al. 2024; Moral and Trapero 2012).

The influence of climate factors on *Colletotrichum* species affecting olives has been explored in some cases. Recently, Brugneti et al. (2024) described the impact of temperature on the germination and growth of *C. fioriniae*, a species dominant in central Italy and California (Moral et al. 2021). However, despite *C. godetiae* being one of the dominant species causing olive anthracnose, there is a notable lack of studies addressing how temperature, humidity, and CO_2_ interact to influence it.

This study aims to deepen our understanding of how climate change may affect the growth of *Colletotrichum godetiae*, the causal agent of olive anthracnose. It was hypothesised that elevated atmospheric CO_2_ concentrations, in interaction with temperature and water activity, would influence the pathogen's mycelial growth and development under simulated climate change conditions. Generating such knowledge is essential for farmers and stakeholders in the olive industry to develop effective disease management strategies and to ensure the long‐term sustainability of olive cultivation.

## 2 Materials and Methods

### 2.1 Fungal Isolates

The *C. godetiae* reference strains Col‐558 and Col‐493 were used throughout the different experiments. This species was selected due to its impact in Spain, where it is the dominant pathogen causing olive anthracnose. Both strains were isolated from infected olive fruits. Both strains are maintained in the mycological collection of the Agronomy Department of the University of Cordoba, Spain. They were previously identified using a multilocus DNA approach (Moral et al. 2021). Before use, the fungi were cultured for 7 days on PDA (Potato Dextrose Agar, Thermo Fisher Scientific, USA) at 21°C.

### 2.2 Media Preparation

Two types of culture media were prepared: commercial PDA (Potato Dextrose Agar, Thermo Fisher Scientific, USA) and ‘Picudo’ fruit‐agar medium. Potato Dextrose Agar (PDA) was used as the reference medium for *Colletotrichum* growth (Moral et al. 2021). In addition, a ‘Picudo’ fruit‐agar medium was developed to evaluate pathogen growth in the presence of host tissue while allowing the combined assessment of temperature and water activity effects. Using whole fruits would have limited this approach, since water activity in olives is fixed and fruit tissues rapidly wrinkle and deteriorate at temperatures above 30°C. The fruit‐agar medium overcomes these constraints by mimicking the physicochemical properties of the olive mesocarp. Homogeneous media were prepared from olive powder obtained under minimal enzymatic degradation: fruits were frozen with liquid nitrogen, then lyophilized, and finally ground in a cryogenic nitrogen mill, as described below.

The ‘Picudo’ fruit‐agar medium was prepared with 2% (w/v) of ‘Picudo’ olive fruit powder and 2% (w/v) of agar. The medium was prepared using olive fruits at ripe state 2, reddish‐purple peel (Barranco et al. 2005), collected in an orchard in Cordoba province (southern Spain). The ‘Picudo’ was selected considering its high susceptibility to the pathogen and its economic importance (Moral et al. 2017). The aw in ‘Picudo’ olive fruits was evaluated with a water activity analyser Rotronic‐HP23‐AW‐A (Vaisala, Vantaa, Finland). The fruits were then frozen in liquid nitrogen and freeze‐dried to a constant weight, confirming that the sample was completely dehydrated. Freeze‐drying was performed with a ScanVac CoolSafe freeze‐dryer (LaboGene A/S, Denmark). The ‘Picudo’ powder was obtained by grinding freeze‐dried fruit samples for 2 min at 5.0 Hz (frequency) with liquid nitrogen using a Retsch CryoMill (Retsch GmbH, Haan, Germany). Subsequently, the ‘Picudo’ fruit‐agar medium was adjusted with glycerol to 0.94 aw to mimic the conditions of the fruits, while the PDA medium was 0.98 aw. The pH levels in media were 5.56 and 5.74 in PDA and ‘Picudo’ fruit‐agar medium, respectively.

This study aimed to establish different environmental conditions. For this purpose, in one of the two experiments conducted, both media (PDA and ‘Picudo’ fruit‐agar medium) were adjusted to different water activity levels (aw = 0.90, 0.94, and 0.98) using glycerol, and the aw was verified with the previously described Rotronic‐HP23‐AW‐A equipment.

Finally, we transferred 5‐mm agar plugs (with the mycelium touching the new media) obtained with a sterile cork borer from the growing margins of the stock culture colony to the center of the Petri dishes.

### 2.3 Incubation Parameters

Petri plates were incubated under control conditions within an airtight chamber with a volume of 12 L (30 × 20 × 20 cm). Each treatment, representing different growth conditions, was separated within the chamber. Two different assays were conducted. In the first one, we studied the effects of four temperatures (10°C, 20°C, 25°C and 35°C) and two concentrations of CO2 (400 and 1000 ppm) on mycelium growth. Eight treatments, resulting from the combination of four temperatures and two CO_2_ conditions, were evaluated in two culture media (PDA and ‘Picudo’ fruit‐agar medium) with five repetitions. The study was conducted with two strains (Col‐558 and Col‐493). In total, 160 petri dishes were used. As mentioned above, the ‘Picudo’ fruit‐agar medium was adjusted to 0.94 aw, while the PDA medium was maintained at 0.98 as control conditions. Airtight chambers were connected to a CO2 source. The CO2 was applied to each incubation chamber through a flowmeter at 3 L/min for 4 min to control the CO2 concentration (Alicat Scientific, Tucson, USA). The CO2 concentration was renewed daily, taking advantage of the moment of measurement. The airtight chamber was opened and aerated to restore the ambient concentration of CO_2_ (400 ppm), while the chambers with 1000 ppm conditions were forced to have a continuous flow of CO2.

In the second assay, the fungal strains were incubated at four temperatures (10°C, 20°C, 25°C and 35°C) and three aw (0.90, 0.94 and 0.98). Twelve treatments, resulting from the combination of four temperatures and three aw conditions, were evaluated in two culture media (PDA and ‘Picudo’ fruit‐agar medium) with five repetitions. The study was conducted with two strains (Col‐558 and Col‐493). In total, 240 petri dishes were used. Each culture medium and incubation chamber was adjusted to the specific aw of the treatment. To achieve this, each airtight chamber contained a 200 mL glass jar filled with glycerol–water solutions, maintaining the equilibrium relative humidity at the target aw level. The glycerol‐water solutions were replaced with fresh solutions every 2 days during the incubation.

In summary, five replicated dishes were used for each combination of factors (strains‐medium‐temperature‐ CO_2_ concentration or strain‐medium‐temperature‐aw). Petri dishes were incubated for 14 days in both assays.

### 2.4 Data Collection and Statistical Analysis

Each Petri dish was assessed once daily at a fixed hour throughout the 14‐day evaluation period. The pathogen's diametric growth was measured using a millimetric ruler according to two perpendicular diameters. Then, the mean diameter growth per day was used in a linear model described by Medina and Magan (2010). For this, a linear regression was extracted. The lag time phase at the beginning and end of incubation was not used to fit the regression. From the equation, the lag phase (λ) was defined as the time required for the pathogen to initiate mycelial growth, and the mycelial growth rates (μmax) were obtained. Lag phase (λ) was determined from the linear equation *y* = *ax* + *b*; solving for *x* when *y* = 5 mm, mycelial growth rates (μmax) are the slope (*a* value). The lag phase (λ) typically ranged from 0 to 3 days under optimal conditions (20°C–25°C, aw ≥ 0.94), and exceeded 14 days at 10°C or 0.90 aw.

Statistical analysis was performed using the software Statistix (analytical software SX, version 10, Tallahassee, FL, USA). Mycelial growth rate data were tested for normality using the Shapiro–Wilk test and homogeneity using Levene's test. Subsequently, a factorial ANOVA was performed independently for each assay. The Tukey's test was used to make an all‐pairwise comparison *p* ≤ 0.05. Additionally, the explained variance was calculated to assess how much of the variability in the dependent variable can be attributed to the independent variables or factors being studied. So, the sums of the squares of each factor and their interactions were calculated to estimate the size effect of the independent variables. For that, we used the sums of each factor's squares and their interactions to estimate the size effect of the independent variables (ր2 = (SS_factor_/SS_Total_) × 100).

## 3 Results and Discussion

### 3.1 Effect of CO_2_ ‐Temperature Interaction on Time Before Pathogen Growth

While various studies have focused on the impact of climate change on different olive parasites (Abd El‐Salam et al. 2019; Requena‐Mullor et al. 2020), less attention has been paid to *Colletotrichum* spp., the principal pathogens of olive fruit. In our study on the effects of temperature, CO_2_, and culture medium on pathogen growth, temperature emerged as the primary factor explaining 64.2% (*F* = 3811.36, *p* < *0.001*) of the total variance in the lag time before growth (λ), followed by the medium (16.2%, *F* = 2884.50, *p* < 0.001) and the interaction medium × temperature (17.9%, *F* = 1065.13, *p* < 0.001) (Table S1). A consistent trend was observed for mycelial growth, as shown in Table S2, where temperature accounted for 49.4% (*F* = 4666.42, *p* < 0.001) of total variation, followed by the medium (29.8%, *F* = 8454.78, *p* < 0.001) and the interaction between medium×temperature (16.7%, *F* = 1575.15, *p* < 0.001). This highlights temperature as the dominant environmental driver of fungal growth dynamics.

In this case, the medium exerted a stronger influence on mycelial growth than on lag time. This pattern may be related to differences in aw, since the medium prepared with olive fruit was adjusted to 0.94 aw to simulate the fruit's natural conditions, whereas PDA medium had a higher value of 0.98 aw. These results suggest a tendency for the pathogen to require higher water availability to maintain active growth over time.

Statistical analysis also indicated that strain differences were statistically detectable but biologically minor (~2% variance); hence, they were treated as negligible in growth rate or lag time between *C. godetiae* strains (Tables S1, S2).

Although CO_2_ is essential for fungal physiology, no significant effects were detected within the tested concentrations (400–1000 ppm) for either lag time (0.01%, *F =* 2.16, *p =* 0.144) or growth rate (0.00%, *F =* 0.16, *p =* 0.691), indicating that CO_2_ alone does not substantially influence the mycelial development of *C. godetiae* under in vitro conditions. This apparent tolerance is noteworthy, given that many fungi exhibit altered growth or virulence under elevated CO_2_, and that microbial systems generally display threshold responses beyond which CO_2_ becomes inhibitory or lethal. In some fungal pathogens, exposure to elevated CO_2_ in laboratory conditions has been associated with mutations and increased virulence, likely due to disruptions in metabolic pathways such as the tricarboxylic acid (TCA) cycle and pyrimidine biosynthesis (Chadwick and Lin 2024). Moreover, early stages of fungal development demand high metabolic activity and generate substantial CO_2_ fluxes (Pavlík et al. 2020), suggesting that carbon availability can influence developmental trajectories. Whether the tolerance observed in vitro for *C. godetiae* is maintained under field conditions, where CO_2_ strongly interacts with plant photosynthesis and host defence responses, or whether long‐term exposure could drive genomic or phenotypic adaptation remains an open question. Overall, these findings contribute to understanding how fungal pathogens may respond to future climate change scenarios.

Considering the significance of the different interactions between factors (Table S1), we separated the temperature and medium variables in the statistical data analysis (Tables 1 and 2). In PDA medium, mycelial growth began within the first 24 h at 20°C and 25°C, while at 10°C, strains started to grow 2.10 days after transfer. The fastest mycelial growth was observed at 25°C and 1000 ppm CO_2_, with minimal lag time. In the case of medium supplemented with olive fruit ‘Picudo’ (‘Picudo’‐Agar), no growth was observed at 10°C and 35°C even after 14 days, whereas growth occurred at 20°C and 25°C. Similarly, Brugneti et al. (2024) reported that mycelial growth of *C. fioriniae* was optimal between 20°C–25°C, with significantly reduced growth at 10°C and no growth at 35°C. Additionally, spore germination rates were highest at 25°C, with a notable decline at a lower temperature. In artificial inoculations of olive fruits ‘Hojiblanca’, *C. godetiae* showed an optimum temperature range for infection of 20°C–24°C (Moral et al. 2012). In epidemiological studies, we have detected that when the mean temperature drops to 7°C under field conditions, the fruit tends to accumulate latent infection of the pathogen without developing symptoms (Moral and Trapero 2012). The fruit susceptibility to the pathogen varies depending on olive genotype (Riolo et al. 2023; Moral et al. 2017), but it is also influenced by other factors, such as fruit ripeness and the presence of wounds (Agosteo et al. 2023). Olive fruits, even those of susceptible genotypes, possess constitutive resistance mechanisms against *Colletotrichum* infection, primarily through phenolic compounds (Miho et al. 2024). Hence, while the PDA medium provides essential nutrients for pathogen growth, the ‘Picudo’ fruit‐agar medium may contain antifungal compounds, such as phenolic compounds or waxes, influencing pathogen growth.

**TABLE 1:** Comparison of mean lag time before growth (λ; in days) of *Colletotrichum godetiae* for two media (Potato Dextrose Agar (PDA) and ‘Picudo’ fruit‐agar) in response to four temperatures (10°C, 20°C, 25°C and 35°C).

Media	CO_2_ (ppm)	Temperature			
		10°C	20°C	25°C	35°C
		λ ± SD	λ ± SD	λ ± SD	λ ± SD
‘Picudo’ fruit‐agar	400	> 14.00	3.89 ± 1.22	3.11 ± 1.79	> 14.00
‘Picudo’ fruit‐agar	1000	> 14.00	4.43 ± 0.60	3.36 ± 0.92	> 14.00
**‘Picudo’**		**> 14.00 a**	**4.16 ± 0.98 b**	**3.24 ± 1.39 c**	**> 14.00 a**
PDA	400	1.91 ± 0.32	0.74 ± 0.08	0.79 ± 0.26	> 14.00
PDA	1000	2.30 ± 0.22	1.06 ± 0.42	0.05 ± 0.14	> 14.00
**PDA**		**2.10 ± 0.34 d**	**0.90 ± 0.34 e**	**0.37 ± 0.48 e**	**> 14.00 a**

*Note:* For each combination of temperature‐medium, values with different letters are statistically different according to the Factorial AOV at Tukey test (*p* ≤ 0.05). The bold values indicate the average values for each medium and temperature.

**TABLE 2:** Comparison of mean mycelial growth rate (mm/day) of *Colletotrichum godetiae* achieved by the linear model for two media (Potato Dextrose Agar (PDA) and ‘Picudo’ fruit‐agar) in response to four temperatures (10°C, 20°C, 25°C, and 35°C).

Media	Temperature			
	10°C mm/day	20°C mm/day	25°C mm/day	35°C mm/day
‘Picudo’ fruit‐agar	0.00 ± 0.00 e	1.75 ± 0.66 d	1.76 ± 0.50 d	0.00 ± 0.00 e
PDA	2.59 ± 0.22 c	7.42 ± 0.69 a	4.07 ± 0.68 b	0.00 ± 0.00 e

*Note:* Values with different letters are statistically different according to the Factorial AOV at Tukey test (*p* ≤ 0.05).

Considering the mycelial growth, the temperature and medium variables were analysed separately due to significant interactions (Table S2). In addition, statistical analysis indicated that differences in growth rate between *C. godetiae* strains were negligible (Table S2). The highest mycelial growth rate of *C. godetiae* was assessed in PDA medium at 20°C, with 7.42 mm/day, followed by 25°C with 4.07 mm/day, and 2.59 mm/day at 10°C. No growth was detected at 35°C (Table 2). In fruit inoculation, we have reported that temperature significantly affects olive fruit infection dynamics, with major differences in pathogenicity observed among *Colletotrichum* species and smaller variations also detected among isolates of the same species (Moral et al. 2012, 2021).

### 3.2 Effect of the Interaction Temperature and Aw in Pathogen Growth

In the second assay, mycelial growth was primarily influenced by aw (38.0%, *F* = 969.65, *p* < 0.001) and temperature (34.2%, *F* = 582.58, *p* < 0.001) (Table S3). When analysing the response for the lag time before mycelial growth (λ), aw also showed a strong influence on this variable, accounting for 49.4% of the variation (*F* = 3411.9, *p* < 0.001), and exerting an even greater effect than on growth rate.

**FIGURE 1:**
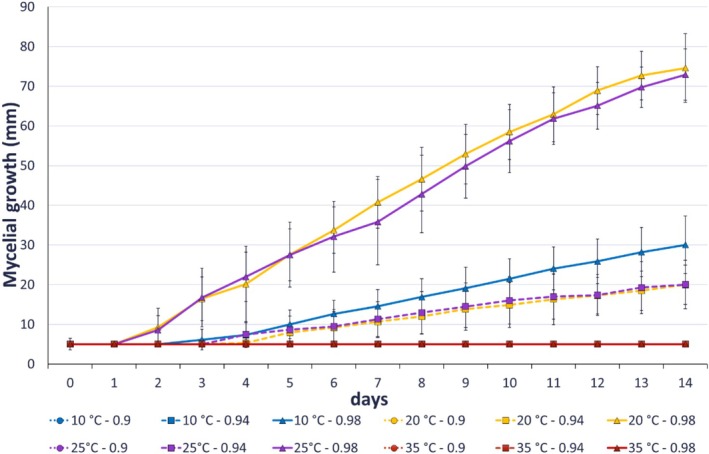
Effect of four temperatures (10°C, 20°C, 25°C and 35°C) and three water activities (aw) (0.90, 0.94 and 0.98) on mycelial growth evolution of *Colletotrichum godetiae* diameter data measured over 14 days. Results are presented as mean values evaluated in two culture media (PDA and ‘Picudo’ fruit‐agar medium) using two strains (Col‐558 and Col‐493). Points represent the mean of five replicated Petri dishes, and bars represent SD (mean ± SD).

When *C. godetiae* strains were cultured at 0.90 aw, no growth was observed at any temperature over 14 days. In the case of 0.94 aw and 20°C, the pathogen showed a lag time of 2.39 days, while at 25°C, it was 1.91 days, although there were no significant differences (*p* > 0.05) between both temperatures (Table 3). At 0.98 aw, *C*. *godetiae* cultures had a lag time of 3.12 days at 10°C, compared to 0.12 and 0.02 days at 20°C and 25°C, respectively. Conversely, ANOVA showed no significant effect of the media type (PDA y ‘Picudo’ fruit‐agar) or interaction between media and temperature or aw (Table S4). Figure 1 and Figure S1 illustrate the growth rate and daily colony diameter over 14 days.

**TABLE 3:** Comparison of mean lag time before mycelial growth (λ; in days) achieved by the linear model in response to four temperatures (10°C, 20°C, 25°C and 35°C) and three water activities (0.90, 0.94 and 0.98).

Temperature	0.90 aw	0.94 aw	0.98 aw
	λ ± SD	λ ± SD	λ ± SD
10°C	> 14.00 a	> 14.00 a	3.12 ± 2.10 b
20°C	> 14.00 a	2.39 ± 1.98 b	0.12 ± 1.28 c
25°C	> 14.00 a	1.91 ± 1.75 b	0.02 ± 1.44 c
35°C	> 14.00 a	> 14.00 a	> 14.00 a

*Note:* Values with different letters are statistically different according to the Factorial AOV at Tukey test (*p* ≤ 0.05).

For extreme xerophilic fungal species, metabolic activity and cell division generally cease when aw drops between 0.70 and 0.64 (Stevenson et al. 2017). *Colletotrichum* species are not xerophilic, requiring high humidity for optimal growth and infection, with rainfall droplets serving as the main dispersal mechanism (Wharton and Diéguez‐Uribeondo 2004). To date, the minimum aw threshold for *C. godetiae* growth has not been established, although in highly resistant olive varieties, the pathogen cannot cause symptoms even under optimal infection conditions (Moral et al. 2017).

When the pathogen was incubated at 0.94 aw, no growth was observed at 10°C or 35°C. At 20°C and 25°C, *C. godetiae* showed a similar, slow growth rate of 1.44 mm/day and 1.40 mm/day, respectively. Growth initiation occurred around days 5 and 6, indicating an extended lag phase under these conditions. Compared to the first assay, the reduced mycelial growth rate is attributed to the aw restriction, as PDA culture in the initial experiment did not impose such limitations. At aw = 0.98, the limiting temperature was significantly lower, with no growth at 35°C and a reduced growth rate of 2.33 mm/day at 10°C. Growth at 20°C and 25°C differed slightly, with 20°C yielding a slightly higher rate than 25°C (5.85 mm/day vs. 5.48 mm/day, respectively). As shown in Figure 1, the final colony diameter of *C. godetiae* at aw = 0.98 was 31.05 mm at 10°C, 75.00 mm at 20°C, and 72.92 mm at 25°C. At aw = 0.94, final growth was significantly lower, reaching only 19.97 mm at 20°C and 20.03 mm at 25°C.

These in vitro results align with field observations, where the fungus primarily survives over summers, probably as an appressorium, on the surface of developing fruit in Mediterranean olive orchards, with summer temperatures exceeding 35°C and minimal rainfall (< 5 mm) (Cacciola et al. 2012; Moral et al. 2009). As temperatures decrease (14°C–25°C) during autumn, humidity becomes more favourable, and antifungal phenolic compounds decrease on mature fruit; the fungus sporulates on fruit (Moral et al. 2009, 2012; Miho et al. 2024). Conde‐Innamorato et al. (2025) demonstrated that reduced irrigation enhances olive fruit resistance to *Colletotrichum*, possibly due to an increased concentration of phenolic compounds and a decrease in aw. Overall, these trends suggest that water availability and temperature strongly shape the epidemiological dynamics of olive anthracnose.

## 4 Conclusion

In the present study, no variation in the growth of the pathogen due to increased CO_2_ levels was detected. However, concluding that CO_2_ will not affect anthracnose development under field conditions is premature, given the complex interactions between the pathogen and its host in a changing environment. Despite using olive fruits of a susceptible cultivar, pathogen mycelial growth was reduced and delayed in medium containing fruit compared to PDA. Activity water was the most limiting factor, suggesting that drought or altered rainfall patterns driven by climate change have a greater impact on anthracnose development than atmospheric CO_2_. Furthermore, *C. godetiae* preferred temperatures around 20°C and stopped growing at 35°C, regardless of aw.

This indicates that the increasingly hot summers projected for Mediterranean regions could suppress active growth or disease development. However, *C. godetiae* is known to oversummer as a melanized appressorium, which remains quiescent on the fruit surface. Thus, while high temperatures may suppress disease development during peak summer, the pathogen's persistence is not necessarily compromised. At the same time, climate change could favour the emergence or spread of more thermotolerant pathogens. By integrating multiple environmental factors, including temperature and water activity, this work provides novel insights into pathogen behaviour under climate change scenarios and improves epidemiological knowledge of the most important olive fruit disease worldwide.

## Author Contributions

**Anabel Expósito‐Díaz:** conceptualization, writing – original draft, writing – review and editing, visualisation, data curation, formal analysis, methodology, investigation. **Juan Moral:** methodology, writing – review and editing, conceptualization, investigation, funding acquisition, resources. **Angel Medina:** methodology, writing – review and editing, conceptualization, investigation, funding acquisition, resources.

## Funding

This work was supported by the Ministerio de Ciencia, Innovación y Universidades (PID2020‐117550RA‐I00).

## Conflicts of Interest

The authors declare no conflicts of interest.

## Supporting information


**Data S1:** Supporting Information.

## Data Availability

The data that support the findings of this study are available on request from the corresponding author. The data are not publicly available due to privacy or ethical restrictions.

## References

[emi470276-bib-0001] Abd El‐Salam, A. M. E. , S. A. W. Salem , R. S. Abdel‐Rahman , H. H. El‐Behery , and M. A. Magd Elden . 2019. “Effects of Climatic Changes on Olive Fly, *Bactrocera oleae* (Rossi) Population Dynamic With Respect to the Efficacy of Its Larval Parasitoid in Egyptian Olive Trees.” Bulletin of the National Research Centre 43: 1–9. 10.1186/S42269-019-0220-9.

[emi470276-bib-0002] Agosteo, G. E. , A. Ragazzi , G. Surico , and S. O. Cacciola . 2023. “Olive Diseases.” In The Olive: Botany and Production, edited by A. Fabbri , L. Baldoni , T. Caruso , and F. Famiani , 565–610. CABI. 10.1079/9781789247350.0023.

[emi470276-bib-0003] Almiman, B. 2024. “Effects of Temperature and Water Activity on 25 de Novo Strains of Pathogenic Plant Fungi in Al‐Baha and Baljurashi Cities in Saudi Arabia.” Journal of Umm Al‐Qura University for Applied Sciences 10: 301–312. 10.1007/s43994-023-00105-x.

[emi470276-bib-0004] Barbosa‐Cánovas, G. V. , A. J. Fontana , S. J. Schmidt , and T. P. Labuza . 2007. Water Activity in Foods: Fundamentals and Applications. Wiley‐Blackwell.

[emi470276-bib-0005] Barranco, D. , L. Rallo , J. M. Caballero , et al. 2005. Variedades de Olivo en España. Junta de Andalucía.

[emi470276-bib-0006] Benlloch‐González, M. , R. Sánchez‐Lucas , M. A. Bejaoui , M. Benlloch , and R. Fernández‐Escobar . 2019. “Global Warming Affects Yield and Fruit Maturation of Olive Trees Growing Under Field Conditions.” Scientia Horticulturae 249: 162–167. 10.1016/j.scienta.2019.01.046.

[emi470276-bib-0007] Brugneti, F. , L. Rossini , M. I. Drais , S. Turco , and A. Mazzaglia . 2024. “Effect of Temperature on In Vitro Germination and Growth of *Colletotrichum fioriniae*, a New Emerging Pathogen of Olive Fruits.” Environmental Microbiology Reports 16, no. 5: e13275. 10.1111/1758-2229.13275. 39228346 PMC11372289

[emi470276-bib-0008] Cacciola, S. O. , R. Faedda , F. Sinatra , et al. 2012. “Olive Anthracnose.” Journal of Plant Pathology 94, no. 1: 29–44.

[emi470276-bib-0009] Calderón, R. , C. Lucena , J. L. Trapero‐Casas , P. J. Zarco‐Tejada , and J. A. Navas‐Cortés . 2014. “Soil Temperature Determines the Reaction of Olive Cultivars to *Verticillium dahliae* Pathotypes.” PLoS One 9: e110664. 10.1371/journal.pone.0110664. 25330093 PMC4201566

[emi470276-bib-0010] Cervini, C. , A. Gallo , L. Piemontese , et al. 2020. “Effects of Temperature and Water Activity Change on Ecophysiology of Ochratoxigenic *Aspergillus carbonarius* in Field‐Simulating Conditions.” International Journal of Food Microbiology 315: 108420. 10.1016/j.ijfoodmicro.2019.108420. 31731232

[emi470276-bib-0011] Chadwick, B. J. , and X. Lin . 2024. “Effects of CO_2_ in Fungi.” Current Opinion in Microbiology 79: 102488. 10.1016/j.mib.2024.102488. 38759247 PMC11162916

[emi470276-bib-0042] Conde‐Innamorato, P. , G. P. García‐Inza , J. Mansilla , et al. 2025. “Moderate Water Stress Improve Resistance to Anthracnose Rot in Arbequina Olive Fruits.” European Journal of Plant Pathology 171, no. 1: 53–65.

[emi470276-bib-0012] FAO . 2024. FAOSTAT. FAOSTAT‐Crops and Livestock Products. https://www.fao.org/faostat/en/#data/QCL/visualize.

[emi470276-bib-0013] Foscolou, A. , E. Critselis , and D. Panagiotakos . 2018. “Olive Oil Consumption and Human Health: A Narrative Review.” Maturitas 118: 60–66. 10.1016/j.maturitas.2018.10.013. 30415757

[emi470276-bib-0014] Habbadi, K. , I. Maafa , A. Benbouazza , et al. 2023. “Differential Response of Olive Cultivars to Leaf Spot Disease (*Fusicladium oleagineum*) Under Climate Warming Conditions in Morocco.” Horticulturae 9: 589. 10.3390/horticulturae9050589.

[emi470276-bib-0015] IPCC . 2019. Global Warming of 1.5°C. https://www.ipcc.ch/site/assets/uploads/sites/2/2019/06/SR15_Full_Report_Low_Res.pdf.

[emi470276-bib-0016] Leoni, C. , J. Bruzzone , J. J. Villamil , et al. 2018. “Percentage of Anthracnose (*Colletotrichum acutatum* s.s.) Acceptable in Olives for the Production of Extra Virgin Olive Oil.” Crop Protection 108: 47–53. 10.1016/j.cropro.2018.02.013.

[emi470276-bib-0017] Lionello, P. , and L. Scarascia . 2018. “The Relation Between Climate Change in the Mediterranean Region and Global Warming.” Regional Environmental Change 18: 1481–1493. 10.1007/s10113-018-1290-1.

[emi470276-bib-0018] Lucena, B. , T. Manrique , and M. A. Méndez . 2017. “La Olivicultura en el Mundo y en España.” In El Cultivo del Olivo, edited by D. Barranco , R. Fernandez‐Escobar , and L. Rallo , 3rd ed., 3–33. Ediciones Mundi‐Prensa.

[emi470276-bib-0019] Lüthi, D. , M. Le Floch , B. Bereiter , et al. 2008. “High‐Resolution Carbon Dioxide Concentration Record 650,000–800,000 Years Before Present.” Nature 453: 379–382. 10.1038/nature06949. 18480821

[emi470276-bib-0020] Magan, N. , A. Medina , and D. Aldred . 2011. “Possible Climate‐Change Effects on Mycotoxin Contamination of Food Crops Pre‐ and Postharvest.” Plant Pathology 60, no. 1: 150–163. 10.1111/j.1365-3059.2010.02412.x.

[emi470276-bib-0021] Medina, A. , M. K. Gilbert , B. M. Mack , et al. 2017. “Interactions Between Water Activity and Temperature on the Aspergillus Flavus Transcriptome and Aflatoxin B1 Production.” International Journal of Food Microbiology 256: 36–44. 10.1016/j.ijfoodmicro.2017.05.020. 28582664

[emi470276-bib-0022] Medina, A. , and N. Magan . 2010. “Comparisons of Water Activity and Temperature Impacts on Growth of *Fusarium langsethiae* Strains From Northern Europe on Oat‐Based Media.” International Journal of Food Microbiology 142: 365–369. 10.1016/j.ijfoodmicro.2010.07.021. 20688410

[emi470276-bib-0023] Miho, H. , A. Expósito‐Díaz , M. I. Marquez‐Perez , et al. 2024. “The Dynamic Changes in Olive Fruit Phenolic Metabolism and Its Contribution to the Activation of Quiescent *Colletotrichum* Infection.” Food Chemistry 450: 30. 10.2139/SSRN.4682830. 38613962

[emi470276-bib-0024] Moral, J. , C. Agustí‐Brisach , M. C. Raya , et al. 2021. “Diversity of *Colletotrichum* Species Associated With Olive Anthracnose Worldwide.” Journal of Fungi 7, no. 9: 741. 10.3390/jof7090741. 34575779 PMC8466006

[emi470276-bib-0025] Moral, J. , R. de Oliveira , and A. Trapero . 2009. “Elucidation of the Disease Cycle of Olive Anthracnose Caused by *Colletotrichum acutatum* .” Phytopathology 99: 548–556. 10.1094/phyto-99-5-0548. 19351251

[emi470276-bib-0026] Moral, J. , J. Jurado‐Bello , M. I. Sánchez , R. De Oliveira , and A. Trapero . 2012. “Effect of Temperature, Wetness Duration, and Planting Density on Olive Anthracnose Caused by *Colletotrichum* spp.” Phytopathology 99: 974–981. 10.1094/phyto-12-11-0343. 22957821

[emi470276-bib-0027] Moral, J. , and A. Trapero . 2012. “Mummified Fruit as a Source of Inoculum and Disease Dynamics of Olive Anthracnose Caused by *Colletotrichum* spp.” Phytopathology 102: 982–989. 10.1094/phyto-12-11-0344. 22957822

[emi470276-bib-0028] Moral, J. , C. Xaviér , L. F. Roca , J. Romero , W. Moreda , and A. Trapero . 2014. “Olive Anthracnose and Its Effect on Oil Quality.” Grasas y Aceites 65: e028. 10.3989/gya.110913.

[emi470276-bib-0029] Moral, J. , C. J. Xaviér , J. R. Viruega , L. F. Roca , J. Caballero , and A. Trapero . 2017. “Variability in Susceptibility to Anthracnose in the World Collection of Olive Cultivars of Cordoba (Spain).” Frontiers in Plant Science 8: 299731. 10.3389/fpls.2017.01892. PMC5681583 29163612

[emi470276-bib-0030] Orlowsky, B. , and S. Seneviratne . 2012. “Global Changes in Extreme Events: Regional and Seasonal Dimension.” Spring 110: 669–696. 10.1007/s10584-011-0122-9.

[emi470276-bib-0031] Pavlík, M. , P. Fleischer , P. Fleischer Jr. , M. Pavlík Jr. , and M. Šuleková . 2020. “Evaluation of the Carbon Dioxide Production by Fungi Under Different Growing Conditions.” Current Microbiology 77: 2374–2384. 10.1007/s00284-020-02033-z. 32472260

[emi470276-bib-0032] Raza, M. M. , and D. P. Bebber . 2022. “Climate Change and Plant Pathogens.” Current Opinion in Microbiology 70: 102233. 10.1016/J.MIB.2022.102233. 36370642

[emi470276-bib-0033] Requena‐Mullor, J. M. , J. M. García‐Garrido , P. A. García , and E. Rodríguez . 2020. “Climatic Drivers of *Verticillium dahliae* Occurrence in Mediterranean Olive‐Growing Areas of Southern Spain.” PLoS One 15: 1–16. 10.1371/journal.pone.0232648. PMC7773261 33378350

[emi470276-bib-0034] Riolo, M. , A. Pane , E. Santilli , S. Moricca , and S. O. Cacciola . 2023. “Susceptibility of Italian Olive Cultivars to Various *Colletotrichum* Species Associated With Fruit Anthracnose.” Plant Pathology 72, no. 2: 255–267. 10.1111/PPA.13652.

[emi470276-bib-0035] Samaniego, L. , S. Thober , R. Kumar , et al. 2018. “Anthropogenic Warming Exacerbates European Soil Moisture Droughts.” Nature Climate Change 8: 421–426.

[emi470276-bib-0036] Seneviratne, S. I. , M. G. Donat , A. J. Pitman , R. Knutti , and R. L. Wilby . 2016. “Allowable CO_2_ Emissions Based on Regional and Impact‐Related Climate Targets.” Perspective 529: 477–483. 10.1038/nature16542. 26789252

[emi470276-bib-0037] Stevenson, A. , P. G. Hamill , A. Medina , et al. 2017. “Glycerol Enhances Fungal Germination at the Water‐Activity Limit for Life.” Environmental Microbiology 19: 947–967. 10.1111/1462-2920.13530. 27631633 PMC5363249

[emi470276-bib-0038] Talhinhas, P. , A. Loureiro , and H. Oliveira . 2018. “Olive Anthracnose: A Yield‐ and Oil‐Quality‐Degrading Disease Caused by Several Species of *Colletotrichum* That Differ in Virulence, Host Preference, and Geographical Distribution.” Molecular Plant Pathology 19: 1797–1807. 10.1111/MPP.12676. 29517840 PMC6638118

[emi470276-bib-0040] Vogel, J. , E. Paton , V. Aich , and A. Bronstert . 2021. “Increasing Compound Warm Spells and Droughts in the Mediterranean Basin.” Weather and Climate Extremes 32: 100312. 10.1016/J.WACE.2021.100312.

[emi470276-bib-0041] Wharton, P. , and J. Diéguez‐Uribeondo . 2004. “The Biology of *Colletotrichum acutatum* .” Anales del Jardin Botánico de Madrid 61: 3–22.

