# Supplementary material for: Influence of Environmental Factors on the Growth of Colletotrichum godetiae, Causal Agents of Olive Anthracnose in Spain
Source: Environ Microbiol Rep. 2026 Feb 12;18(1):e70276. doi: 10.1111/1758-2229.70276 (PMC12895466; doi:10.1111/1758-2229.70276)
Supplement: Supplementary file 1 — Data S1: Supporting Information. [file EMI4-18-e70276-s001.docx]

**Influence of environmental factors on the growth of *Colletotrichum godetiae*, causal agents of Olive Anthracnose in Spain**

**SUPPLEMENTARY MATERIAL**

**Supplementary table 1.** Analysis of Variance of temperature, culture media, and CO2 concentration on lag time before mycelial growth (λ) of *Colletotrichum godetiae*

|  | | | | | | **Variance explained** |
| --- | --- | --- | --- | --- | --- | --- |
| **Source** | **DF** | **SS** | **MS** | **F** | **P** | **(%)** |
| Repetition | 4 | 0.26 | 0.065 |  |  |  |
| Temperature | 3 | 2373.39 | 791.13 | 3811.36 | 0.000 | **64.15** |
| CO2 | 1 | 0.45 | 0.448 | 2.16 | 0.144 | 0.01 |
| Medium | 1 | 598.74 | 598.74 | 2884.5 | 0.000 | **16.18** |
| Strain | 1 | 8.59 | 8.59 | 41.38 | 0.000 | 0.23 |
| Temperature×CO2 | 3 | 2.75 | 0.918 | 4.42 | 0.005 | 0.07 |
| Temperature×Medium | 3 | 663.27 | 221.09 | 1065.13 | 0.000 | **17.03** |
| Temperature×Strain | 3 | 9.33 | 3.111 | 14.99 | 0.000 | 0.25 |
| CO2×Medium | 1 | 0.71 | 0.712 | 3.43 | 0.066 | 0.02 |
| CO2×Strain | 1 | 2.19 | 2.192 | 10.56 | 0.002 | 0.06 |
| Medium×Strain | 1 | 13.71 | 13.707 | 66.04 | 0.000 | 0.37 |
| Error | 127 | 26.36 | 0.208 |  |  | 0.71 |
| Total | 149 | 3699.75 |  |  |  |  |

Note: SS are marginal (type III) sums of squares

Grand Mean 6.5767

CV 6.93

**Supplementary table 2.** Analysis of variance of temperature, culture media, and CO_2_ concentration on the mycelial growth rate (mm/day) of *Colletotrichum godetiae.*

|  |  |  |  |  |  | **Variance explained** |
| --- | --- | --- | --- | --- | --- | --- |
| **Source** | **DF** | **SS** | **MS** | **F** | **P** | **(%)** |
| Repetition | 4 | 0.431 | 0.108 |  |  |  |
| Temperature | 3 | 345.33 | 115.11 | 4666.42 | 0.000 | **49.37** |
| CO2 | 1 | 0.004 | 0.004 | 0.16 | 0.691 | 0.00 |
| Medium | 1 | 208.56 | 208.56 | 8454.78 | 0.000 | **29.82** |
| Strain | 1 | 14.062 | 14.062 | 570.06 | 0.000 | 2.01 |
| Temperature×CO2 | 3 | 0.043 | 0.014 | 0.59 | 0.625 | 0.01 |
| Temperature×Medium | 3 | 116.566 | 38.855 | 1575.15 | 0.000 | **16.67** |
| Temperature×strain | 3 | 10.847 | 3.616 | 146.58 | 0.000 | 1.55 |
| CO2×Medium | 1 | 0.005 | 0.005 | 0.2 | 0.653 | 0.00 |
| CO2×strain | 1 | 0.064 | 0.064 | 2.58 | 0.111 | 0.01 |
| Medium*strain | 1 | 0.401 | 0.401 | 16.25 | 0.000 | 0.06 |
| Error | 127 | 3.133 | 0.025 |  |  |  |
| Total | 149 | 699.446 |  |  |  |  |

Note: SS are marginal (type III) sums of squares

| Grand Mean | 2.20 |
| --- | --- |
| CV | 7.14 |

**Supplementary table 3**. Analysis of Variance of temperature, culture media, and water activity (aw) on mycelial growth rate (mm/day) of *Colletotrichum godetiae.*

|  | | | | | | **Variance explained** |
| --- | --- | --- | --- | --- | --- | --- |
| **Source** | **DF** | **SS** | **MS** | **F** | **P** | **(%)** |
| Repetition | 4 | 0.21 | 0.05 |  |  |  |
| Temperature | 3 | 2065.97 | 688.66 | 582.58 | 0.000 | **34.24** |
| Aw | 2 | 2292.41 | 1146.21 | 969.65 | 0.000 | **37.99** |
| Medium | 1 | 11.56 | 11.56 | 9.78 | 0.002 | 0.19 |
| Temperature×Aw | 6 | 1482.48 | 247.08 | 209.02 | 0.000 | **24.57** |
| Temperature×Medium | 3 | 4.76 | 1.59 | 1.34 | 0.263 | 0.08 |
| Aw×Medium | 2 | 14.11 | 7.05 | 5.97 | 0.003 | 0.23 |
| Error | 138 | 163.13 | 1.18 |  |  |  |
| Total | 159 | 6034.63 |  |  |  |  |

Note: SS are marginal (type III) sums of squares

| Grand Mean | 8.815 |
| --- | --- |
| CV | 12.33 |

**Supplementary table 4**. Analysis of Variance of the effects of culture media, and water activity (aw) on lag time before mycelial growth (λ) of *Colletotrichum godetiae.*

|  | | | | | | **Variance explained** |
| --- | --- | --- | --- | --- | --- | --- |
| **Source** | **DF** | **SS** | **MS** | **F** | **P** | **(%)** |
| Repetition | 4 | 0.073 | 0.02 |  |  |  |
| Temperature | 3 | 161.09 | 53.70 | 1162.79 | 0.000 | **25.24** |
| Aw | 2 | 315.12 | 157.56 | 3411.88 | 0.000 | **49.38** |
| Medium | 1 | 1.69 | 1.69 | 36.63 | 0.000 | 0.27 |
| Temperature×Aw | 6 | 152.11 | 25.35 | 548.97 | 0.000 | **23.83** |
| Temperature×Medium | 3 | 0.92 | 0.31 | 6.61 | 0.000 | 0.14 |
| Aw×Medium | 2 | 0.88 | 0.44 | 9.53 | 0.000 | 0.14 |
| Error | 137 | 6.33 | 0.05 |  |  |  |
| Total | 158 | 638.21 |  |  |  |  |

Note: SS are marginal (type III) sums of squares

Grand Mean 1.3832

CV 15.54


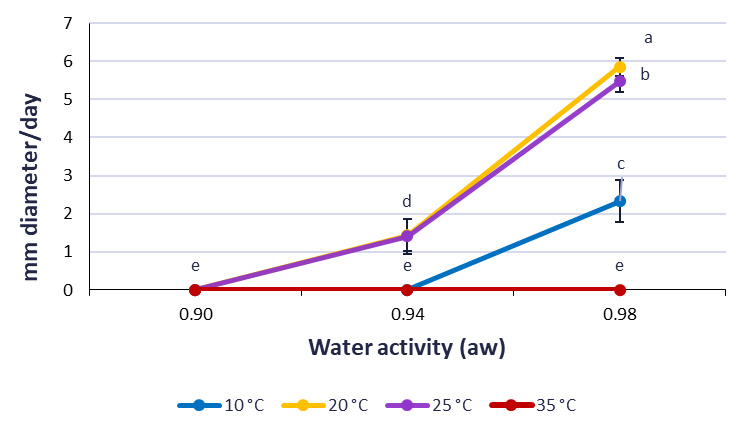


**Supplementary Fig 1**. Effect of three water activities (aw) (0.90, 0.94, and 0.98) and four temperatures (10, 20, 25, and 35°C) on the maximum growth rate of *Colletotrichum godetiae* (µmax; mycelial growth mm /day). Results are presented as mean values evaluated in two culture media (PDA and 'Picudo' fruit-agar medium) using two strains (Col-558 and Col-493). The mean of 5 replicated Petri dishes was measured over 14 days.
